# Analysis of costs for pandemic management in a tertiary-care hospital in Italy: An investment for a more resilient structure

**DOI:** 10.1017/ice.2020.1311

**Published:** 2020-11-04

**Authors:** Angelo Baggiani, Silvia Briani, Grazia Luchini, Mauro Giraldi, Jacopo Fernandez, Carla Collecchi, Matteo Filippi, Lucia Trillini, Domenica Mamone, Maria Carola Martino, Antonella Ciucci, Michele Cristofano, Antonella De Vito, Gabriella Pellegrini, Grazia Valori, Guglielmo Arzilli, Daniele Sironi, Tommaso Mariotti, Francesca Papini, Virginia Casigliani, Giuditta Scardina, Giacomo Visi, Beatrice Casini, Andrea Porretta, Michele Totaro, Gaetano Privitera, Carlo Milli

**Affiliations:** 1 The Azienda Ospedaliero Universitaria Pisana, Pisa, Italy; 2 Department of Translational Research and the New Technologies in Medicine and Surgery, University of Pisa, Pisa, Italy

*To the Editor*—The tertiary-care teaching hospital of Pisa (Azienda Ospedaliera Universitaria Pisana, AOUP) received its first coronavirus disease 2019 (COVID-19) patient on March 4. During the outbreak, this hospital setting underwent major reorganization to respond to the situation. Hospital wards and patient management protocols were modified to quickly adapt to the clinical needs of the patients as well as to ensure patient safety and healthcare worker (HCW) protection from infection.^1,2^

The execution of this strategy to deal with the epidemic imposed significant costs on our hospital. Currently, many expenditure models have been published, as data concerning national health insurance and losses in terms of investments in the various economic sectors.^3-5^

We quantified economic costs incurred by the AOUP from March to July 2020 for the management of the COVID-19 pandemic, focusing on the structural costs for the implementation of the present structure and for the realization of a new COVID-19 hospital.

AOUP is organized into 2 main facilities for a total of 1,082 beds. For pandemic management, the Cisanello facility was reorganized and 160 bed places were dedicated to COVID-19 patients in medical wards, 39 beds in intensive care units, and 24 bed for C-PAP therapy.^1^ Santa Chiara facility is an old pavilion hospital that has been gradually disused. A building that used to accommodate the old emergency room has been renovated as the city’s COVID-19 center.

For the purpose of this analysis, we considered 3 phases: phase 1 (March 4–May 4); phase 2 (May 4–July 4), and phase 3 (March 15–July 4).

In the first phase, the emergency was addressed by increasing response to COVID-19 patients. Ordinary wards were progressively closed to dedicate these areas to COVID-19 patients. Therapeutic pathways were reorganized, and COVID-19 cases were stratified into patients requiring low-, medium-, and high-intensity care. Construction of negative pressure chambers in operating theatres and the extension of intensive care wards were carried out.

In the second phase, the objectives were the radical sanitization of the areas dedicated to COVID-19 patients, the reconversion to their original use, and the gradual resumption of outpatient activities by eliminating the waiting lists created and of visits to patients.

The objective of the third phase was to create a hospital for COVID-19 patients in the Santa Chiara facility to prepare for future outbreaks.

For each phase, we analyzed costs directly related to the preparedness and the management of the epidemic (Table 1).

**Table 1.** Costs Directly Related to the Preparedness and the Management of the COVID-19 Epidemic in Azienda Ospedaliera Universitaria Pisana

Phase	Description	Costs	
		Euros	US$^a^
Phase 1	Technical area (COVID hospital rearrangement)	338,444.51	395,239.17
	Technical area (site safety charges)	4,000.00	4,671.24
	Ventilation system adaption (from positive to negative pressure)	70,000.00	81,755.40
	Health management units (cleaning setting improvement)	154,648.22	180,618.96
	Health management units (biological materials transport)	20,000.00	23,358.69
	Health management units (security services)	65,268.00	76,228.74
	Health management units (special waste disposal)	20,000.00	23,358.69
	Medical devices (including FFP2/FFP3 masks)	1,586,505.57	1,852,829.24
	Health furnishing	633,000.00	739,260.50
	Pharmaceutical spending for COVID patients)	446,666.67	521,647.75
	Pharmaceutical spending for medical gases	233,333.33	272,502.55
	Hydroalcoholic gel	40,000.00	46,714.72
	SARS COV-2 screening test	1,466,666.67	1,712,824.47
	Administrative burden expense (notebook)	20,000.00	23,358.69
	New healthcare workers recruitment	585,136.49	683,342.79
	Accommodation services for employees	10,000.00	11,678.35
Phase 2	Technical areas (aeraulic facilities reconversion and cleaning)	740,000.00	864,197.80
	Health Management Units (wards cleaning and sanitizing)	560,000.00	653,984.76
	Environmental monitoring and microbiological analysis	13,672.00	15,966.57
Phase 3	New COVID hospital reconstruction (structural costs and health furnishing)	3,817,882.00	4,458,636.85
	Environmental monitoring and microbiological analysis	6,882.00	8,037.01

Note. FFP, filtering face piece.

a Exchange rate: 1 euro = 1.1678 USD.

In phase 1, the final cost accrued was €342,444.91 (US$399,952.17) for the technical area. Furthermore, €70,000.00 (US$81,755.20) was attributed to the adaptation of the ventilation system of the wards from positive pressure to negative pressure. For the costs sustained by the health management unit (HMU), we estimated the total expenditure to be €2,479,421.79 (US$2,895,794.65). In total, €1,586,505.57 (US$1,852,929.72) was attributed to medical devices. For pharmaceuticals, €680,000.00 (US$794,119.19) was spent, and €40,000.00 (US$46,712.89) was spent to purchase hydroalcoholic gel for the entire hospital. Screening of patients and HCWs for severe acute respiratory coronavirus virus 2 (SARS-CoV-2) cost €1,466,666.67 (US$1,712,806.10). In addition, €585,136.49 (US$683,346.90) was spent to recruit healthcare workers, and €10,000.00 (US$11,678.42) was spent in accommodation services for employees.

In phase 2, €740,000.00 (US$864,202.99) was spent on the technical area for cleaning and reconversion of the aeraulic facilities. In addition, €560,000.00 (US$653,991.45) was spent by the HMU for cleaning and sanitizing COVID-19 wards. Costs related to environmental monitoring and microbiological analyses performed before the reopening of the departments and restart of ordinary activities amounted to €13,672.00 (US$15,972.23).

In phase 3, €3,817,882.00 (US$4,460,216.00) was spent for the construction of the new COVID-19 patients hospital. Most of this expense was for structural costs and health furnishings. In addition, €6,882.00 (US$8,039.85) was spent for environmental microbiological analyses.

In total, €13,556,355.43 (US$15,837,124.73) was spent globally for the 3 phases: €8,417,919.43 (US$9,837,164.61) was spent in phase 1, €1,313,672.00 (US$1,535,154.60) was spent in phase 2, and €3,824,764.00 (US$4,469,611.93) was spent in phase 3.

Healthcare facilities had to quickly adapt; they underwent huge changes to protect HCWs, to manage COVID-19 patients, and to limit the risk of infection for other inpatients. The way health services are delivered has been greatly modified at our institution during the pandemic.^6^

Meeting these challenges has entailed significant expenditure in economic terms. Even if many reports on the costs that health systems had to face because of the COVID-19 emergency have already been published, it is difficult to quantify the real expenditure, and the estimates are most likely underrepresented. In Italy, according to the ALTEMS report,^7^ the total impact on hospital expenditures has been €1,586,858,655 (US$1,854,400,002).

In our study, the entire process of conversion (the reconversion of Cisanello and requalification of Santa Chiara) cost a total of €13,556,355.43 (US$15,840,470.55). These resources were deployed by making decisions toward significant benefits. Furthermore, we set up adequate infrastructures to meet possible future waves.

The placement of these funds is in accordance with the WHO guidelines, which affirm that expenditures for COVID-19 should lead to longer-term, wider benefits in line with national needs for sustainable capacities.^8^ Our hospital has made efforts and incurred costs to adapt in a short time to the emerging crisis. We used this opportunity to increase the hospital’s resilience and preparedness. The strategies adopted, with the related costs, have allowed us not only to overcome the acute phase but also to prepare the necessary resources for future crises.
